# Factors associated with psychopathological symptoms among nurses at a university hospital

**DOI:** 10.1590/0034-7167-2022-0075

**Published:** 2022-11-28

**Authors:** Sheila Janaína Oliveira Araújo Lima, Danielle Christine Moura dos Santos, Maria de Fátima de Souza Santos, Felicialle Pereira da Silva, Darine Marie Rodrigues da Silva, Jael Maria de Aquino

**Affiliations:** I Universidade de Pernambuco. Recife, Pernambuco, Brazil; II Universidade Federal de Pernambuco. Recife, Pernambuco, Brazil

**Keywords:** Mental Health, Nursing, Occupational Health, Quality of Life, Psychopathology, Salud Mental, Enfermería, Salud Laboral, Calidad de Vida, Psicopatología, Saúde Mental, Enfermagem, Saúde do Trabalhador, Qualidade de Vida, Psicopatologia

## Abstract

**Objectives:**

to analyze the factors associated with psychopathological symptoms among nurses at a university hospital.

**Methods:**

a cross-sectional study developed at a university hospital in Pernambuco, in which 90 nurses participated. A questionnaire with social and professional characteristics and the Symptom Assessment Scale were used.

**Results:**

an association was found between the variable changing profession and the presence of characteristic symptoms of psychoticism, somatization and anxiety among nurses.

**Conclusions:**

the emergence of psychopathological symptoms has influenced nurses’ desire to search for another profession. This evidence reinforces the need for greater investments in better working conditions and welcoming actions in the work environment, in order to provide a better quality of professional life and promote the health of these workers.

## RESUMO

**Objetivos:**

analisar os fatores associados aos sintomas psicopatológicos entre enfermeiros de um hospital universitário.

**Métodos:**

estudo transversal, desenvolvido em um hospital universitário de Pernambuco, em que participaram 90 enfermeiros. Foi utilizado um questionário com características sociais e profissiográficas e a Escala de Avaliação de Sintomas.

**Resultados:**

foi constatada a associação entre a variável mudar de profissão e a presença de sintomas característicos de psicoticismo, somatização e ansiedade entre os enfermeiros.

**Conclusões:**

o surgimento de sintomas psicopatológicos tem influenciado no desejo de enfermeiros na busca por outra profissão. Essa evidência reforça a necessidade de maiores investimentos em melhores condições de trabalho e ações de acolhimento no ambiente laboral, a fim de proporcionar uma melhor qualidade de vida profissional e favorecer a promoção a saúde desses trabalhadores.

## RESUMEN

**Objetivos:**

analizar los factores asociados a síntomas psicopatológicos en enfermeros de un hospital universitario.

**Métodos:**

estudio transversal, desarrollado en un hospital universitario de Pernambuco, en el que participaron 90 enfermeros. Se utilizó un cuestionario con características socioprofesionales y la Escala de Evaluación de Síntomas.

**Resultados:**

se encontró asociación entre la variable cambio de profesión y la presencia de síntomas característicos de psicoticismo, somatización y ansiedad entre los enfermeros.

**Conclusiones:**

la aparición de síntomas psicopatológicos ha influido en el deseo de los enfermeros de buscar otra profesión. Esta evidencia refuerza la necesidad de mayores inversiones en mejores condiciones de trabajo y acciones de acogida en el ambiente de trabajo, con el fin de proporcionar una mejor calidad de vida profesional y promover la salud de estos trabajadores.

## INTRODUCTION

Understanding mental health does not go beyond the simple fact of the absence of disease, but, in a broader sense, incorporates an individual’s physical, social and emotional well-being. Thus, damages to mental health are related to factors such as unhealthy lifestyles, poor working conditions, gender discrimination, social changes, among others^(1)^. For nursing professionals, this concept becomes more complex, due to the continuous imposed tensions and characteristics of the profession, the work environment and the feminization of the profession, which culminates in the double shift (work/home), interfering with the degree of exhaustion and family relationships^(2)^.

Regarding the quality of life at work, it is understood that there are gaps in the concept, as it involves several aspects, such as motivation, satisfaction, working conditions, in addition to personal factors. Thus, intrinsic and extrinsic conditions represent an inference in what is conceived as well-being in the work environment, while this concept of well-being and the relationship of pleasure can be in agreement with positive emotions in the work environment, involving the human being’s understanding of competences and life goals. Thus, it is possible to reflect that the environment both interferes with workers’ life and well-being and suffers interference from the external behaviors and influences that each worker tends to take with them^(3)^.

In this regard, the influence of some issues on working conditions is observed, determining the health-disease process in the work scenario. As an example, we cite working hours spent carrying out activities, shift work and mealtime mismatches that, when incorporated into the demanding factor, exert a certain impact both physically and emotionally, due to the increase in work pace and intensity. Such situations are constant presence in the routine of professionals, especially health professionals, so that they require discussions about the relationship between work and workers’ health^(4)^.

In 2020, the World Health Organization (WHO), in partnership with the International Council of Nurses (INC) and the Nursing Now campaign, issued the report entitled The State of the World’s Nursing 2020, However, it pointed out the precariousness of the work of these professionals, by disclosing the global deficit and its distribution in Brazil and in the world^(5)^.

Concerned with this scenario, the WHO estimated a growth of 15% in mental disorders by 2020, predicting the second cause of absence from work worldwide, and, in Brazil, the third cause of incapacity for work^(6)^. Added to the design of the precarious work profile, there is the history of these professionals in the face of the main pandemics, whose role is highlighted. In 2020, with the advent of COVID-19, the American Nurses Association estimated the participation of 3.8 million nurses in the United States and more than 20 million nurses worldwide, corresponding to the highest percentage of professionals positioned on the front line^(7)^.

In this regard, psychological distress among nursing professionals has become synonymous with disquiet among the scientific community and concern within the scope of people management in health institutions. The influence of the works cenario weaknesses, along with the factors that do not depend on workers’ competence and commitment, should trigger the process of vulnerability among nurses and psychological disorders^(8-9)^.

Studies point to the need to strengthen relationships between job satisfaction, quality of life at work and workers’ health, considering that the quality of nursing care is inseparable from the physical and psychological health conditions of these professionals^(10-12)^.

Given the historical context of nursing, aggravated by the pandemic situation experienced by these professionals, recognizing the factors associated with the illness process favors short and long-term intervention plans, which need to be designed for the sake of nursing professionals’ and society’s health.

## OBJECTIVES

To analyze the factors associated with psychopathological symptoms among nurses at a university hospital.

## METHODS

### Ethical aspects

This study complied with the ethical aspects involved in research with human beings, according to the recommendations of Resolution 466/12 of the Brazilian National Health Council, and was approved by the Research Ethics Committee of the HUOC-PROCAPE-UPE.

### Study design, period and location

This is a descriptive cross-sectional study, guided by the Strengthening the Reporting of Observational Studies in Epidemiology (STROBE) recommendations. Participants were nurses working in a university hospital in the state of Pernambuco, Recife, Brazil, a reference in the care of patients with cardiovascular diseases in northern and northeastern Brazil^(13)^. Data collection took place from May to July 2020.

### Population and sample, inclusion and exclusion criteria

Of the 159 nurses, 90 agreed to participate in the study, and the sample was chosen by convenience. Nurses from the different sectors of the hospital were included, who were part of the effective staff and fit in day and night shifts in managerial activity and direct patient care. Those with less than one year of professional experience and on leave due to vacation, health leave, paid leave or maternity leave during the period of data collection were excluded from the study.

### Study protocol

The approach to the participants took place during the shifts, by invitation, after explanation of the study objectives by the responsible researcher. We guarantee free participation in the study to the recruited nurses, preceded by the signing of the Informed Consent Form (ICF), in two copies. However, the data collection procedure took place in the period classified as “peak of the pandemic”, in which some adaptations were necessary, as the application of the instruments used took place at the workplace, following the safety measures for COVID-19, under the supervision of the main researcher. As a proposal to mitigate possible indications of conflicts of interest, the instruments answered were packaged in unidentified envelopes, in order to contribute to the guarantee of sample secrecy and confidentiality. Thus, data collection was performed, varying between the beginning and end of each work shift, with the presence of participant and the researcher.

Data collection occurred through the application of two instruments: a questionnaire prepared by the researchers themselves and the Symptom Assessment Scale (EAS-40). The first contemplated the social and professional characteristics of nurses’ work, covering the variables: sex; race/color; age group; education; marital status; number of children; service time; sector of activity; employment relationships; weekly workload; working time in the institution; shift; remuneration; work schedule; extra hour; fold on duty; payroll deduction; time spent commuting between home and work; rest time during the shift; hours of sleep per day; sleep quality; use of medication regularly; and having a disease. The second was a self-report instrument, originated by Symptom Checklist-90 (SCL-90-R), prepared by Derogatis^(14)^ and adapted and validated for Brazil^(15)^, which allowed estimating the severity of psychological symptoms of the studied population.

The EAS-40 presents 40 items distributed in four dimensions, with ten items each: 1. Psychoticism, representing continuous between psychosis and depression with symptoms of hostility and paranoids; 2. Obsessiveness/compulsiveness, composed of symptoms of repeated thoughts and actions, accompanied by discomfort in interpersonal relationships; 3. Somatization, which encompasses symptoms of somatic and somatoform disorders; and 4. Anxiety, which ranges from generalized anxiety symptoms to more specific anxiety symptoms, such as phobias.

### Data analysis

The instrument’s correspondence analysis indicated that the intensity of symptoms assessed, at intermediate levels (1 = a little bit, 2 = moderately, 3 = quite a bit), was not very discriminating. From this result, the response options were restricted to three options, distributed in a Likert-type scale, with the following variation: 0 = not at all, 1 = a little bit and 2 = quite a bit^(15)^.

Although this indication contradicts the indication in the literature that scales with less than five points should be avoided^(16)^, the discrimination of three points of severity of symptoms is sufficient for psychological and medical guidance, in order to favor the way patients/individuals perceive symptoms^(15)^.

As for data analysis, a database was built in the program EPI INFO^®^, version 3.5.2, in which double typing was performed. After validity, the database was exported to the Statistical Package for the Social Sciences^®^ (SPSS), version 13.0 for Windows, and Excel 2019, for analysis. The tests were applied with 95% confidence, and the results were presented in a table, with the respective absolute and relative frequencies.

The numerical variables are represented by central tendencies and dispersion measures. In the presence of association, the chi-square test was applied for categorical variables, and the Kolmogorov-Smirnov normality test for quantitative variables. Regarding the comparison between two groups: Student’s t-test (normal distribution) and Mann-Whitney test (non-normal). For comparisons with more than two groups, ANOVA (normal distribution) and Kruskal-Wallis (non-normal), P < 0.05 values were considered significant.

The general symptom index was calculated from the mean of all EAS-40 items and, to calculate the symptoms index in the subscales, the mean of the items in each dimension was obtained. It is emphasized that the closer to 2, the higher the symptom index in each subscale, determining in which dimension the individuals are more symptomatic^(15)^.

## RESULTS

Participation was voluntary, and it was not possible to distribute the population equally between shifts and sectors. Thus, it was found, from the variables studied, that the population was characterized as 90% being female, 56.3% in the age group older than 45 years to 60 years, 42.2% of brown color, 54 .4% married and 71.1% with children.

Still on the professional characteristics of participants, it was found that 60% were involved in direct patient care; 86.7% had specialization as their highest degree; 77.8% had two employment relationships; 46.7% had a weekly workload of between 40 and 60 hours; 52.2% work during the day and 35.6% received a monthly income greater than five minimum wages.

When observing the characteristics related to job satisfaction and lifestyle of these professionals, it was found that 68.9% did not express interest in changing jobs, and this percentage becomes even more expressive regarding the lack of interest in changing professions (74.4%). Subsequently, 56.7% reported having some kind of rest during the shift, and 80.1% reported sleeping between 6 and 8 hours/day and 36.7% considered the quality of this sleep to be regular.

Table 1 presents the percentage of participants’ answers to the 40 questions presented by the Symptom Diagnosis Scale^(15)^. In this, the nurses’ response profile was described based on questions that comprised three possibilities: not at all, a little bit and quite a bit. An increasing degree of intensity was varied for each of the aspects treated, in order to represent participants’ perception by self-report, about the symptoms presented in the respective situations in the four dimensions: psychoticism, obsessiveness/compulsiveness, somatization and anxiety.

**Table 1:** Percentage of answers to the 40 questions assessed by the Symptom Assessment Scale, Recife, Pernambuco, Brazil, 2020

How worried you are		Not atall	A little bit	Quite a bit
**Dimensions**	**Variables**	**Population distribution n (%)**		
Psychocytism	1- Weakness or dizziness	41 (45.5)	42 (46.7)	7 (7.8)
	2- Pains in heart or chest	62 (68.9)	19 (21.1)	9 (10.0)
	3- Feeling afraid in open spaces or on the streets	66 (73.3)	18 (20.0)	6 (6.7)
	4- Thoughts of ending your life^*^	81 (90.0)	3 (3.3)	5 (5.5)
	5- Suddenly scared for no reason	61 (67.8)	24 (26.7)	5 (5.5)
	6- Feeling afraid to go out of the house alone	74 (82.2)	12 (13.3)	4 (4.4)
	7- Pains in lower back	15 (16.7)	39 (43.3)	36 (40.0)
	8- Feelings of worthlessness	56 (62.2)	25 (27.7)	9 (10.0)
	9- Feeling fearful	46 (51.1)	36 (40.0)	8 (8.9)
	10- Nausea or upset stomach	47 (52.2)	36 (40.0)	7 (7.8)
Obsessiveness/compulsiveness	11-Soreness of your muscles	19 (21.1)	40 (44.4)	31 (34.4)
	12-Feeling that you are watched or talked about by others^*^	64 (71.1)	23 (25.5)	2 (2.2)
	13-Having to check and double-check what you do	45 (50.0)	32 (35.5)	13 (14.4)
	14-Feeling afraid to travel on buses, subways, trains	71 (78.9)	13 (14.4)	6 (6.7)
	15-Trouble getting your breath	61 (67.8)	20 (22.2)	9 (10.0)
	16-Hot or cold spells	66 (73.3)	17 (18.9)	7 (7.8)
	17-Having to avoid certain things, places, or activities because they frighten you	61 (67.8)	19 (21.1)	10 (11.1)
	18-Your mind going blank	22 (24.4)	48 (53.3)	20 (22.2)
	19-Numbness or tingling in parts of your body	45 (50.0)	28 (31.1)	17 (18.9)
	20-Feeling hopeless about the future	48 (53.3)	32 (35.5)	10 (11.1)
Somatization	21-Trouble concentrating	23 (25.5)	53 (58.9)	14 (15.5)
	22-Feeling weak in parts of your body	48 (53.3)	35 (38.9)	7 (7.8)
	23-Feeling tense or keyed up	27 (30.0)	48 (53.3)	15 (16.7)
	24-Heavy feelings in your arms or legs	43 (47.8)	38 (42.2)	9 (10.0)
	25-Feeling uneasy when people are watching or talking about you	46 (51.1)	35 (38.9)	9 (10.0)
	26-Having to repeat the same actions such as touching, counting, washing	68 (75.5)	18 (20.0)	4 (4.4)
	27-Having urges to break or smash things	77 (85.5)	11 (12.2)	2 (2.2)
	28-Feeling shy or uneasy with the opposite sex	57 (63.3)	26 (28.9)	7 (7.8)
	29-Feeling uneasy in crowds, such as shopping or at a movie	69 (76.7)	17 (18.9)	4 (4.4)
	30-Feeling everything is an effort	64 (71.1)	22 (24.4)	4 (4.4)
Anxiety	31-Spells of terror or panic	74 (82.2)	11 (12.2)	5 (5.5)
	32-Getting into frequent arguments	64 (71.1)	23 (25.5)	3 (3.3)
	33-Feeling nervous when you are left alone^*^	78 (86.7)	10 (11.1)	1 (1.1)
	34-Feeling lonely even when you are with people	66 (73.3)	22 (24.4)	2 (2.2)
	35-Feeling so restless you couldn’t sit still	65 (72.2)	19 (21.1)	5 (5.5)
	36-Shouting or throwing things	83 (92.2)	5 (5.5)	2 (2.2)
	37-Feeling afraid you will faint in public	79 (87.8)	11 (12.2)	0
	38-Never feeling close to another person	79 (87.8)	8 (8.9)	3 (3.3)
	39-Feelings of guilt	56 (62.2)	28 (31.1)	6 (6.7)
	40-The idea of something is wrong with your mind	60 (66.7)	25 (27.8)	5 (5.5)

(*) Questions not answered by participants.

It is noteworthy that three questions were not answered by three different participants, but they belonged to different dimensions: thoughts of ending your life (psychoticism dimension), feeling that you are watched or talked about by others (obsessiveness/compulsiveness dimension) and feeling nervous when you are left alone (anxiety dimension), but these gaps do not invalidate the result scores.

Table 2 shows the survey participants’ score for the 40 items of the EAS-40, considering the mean and standard deviation of each dimension. The general symptom index (GSI) of EAS-40 was 0.458. Regarding the index of symptoms in the subscales, 0.497 was observed for the first dimension, 0.581 for the second dimension, 0.503 for the third dimension and 0.252 for the fourth dimension, whose highest symptom index close to 2 was 0.581, representing the obsessive/compulsive dimension as the most symptomatic.

**Table 2:** Distribution of means and standard deviation of the Symptom Assessment Scale, according to the general sample and dimensions, Recife, Pernambuco, Brazil, 2020

Variables	Mean ± SD	Median (Q1; Q3)	Minimum - Maximum
Age	47.22 ± 8.99	47.00 (41.00;53.00)	32.00 - 67.00
Score (General index)	0.46±0.33	0.36 (0.20;0.63)	0.00 - 1.43
EAS-1 Psychoticism	0.50±0.38	0.40 (0.20;0.70)	0.00 - 1.60
EAS-2 Obsessiveness/compulsiveness	0.58±0.41	0.50 (0.30;0.80)	0.00 - 1.90
EAS-3 Somatization	0.50±0.37	0.40 (0.20;0.73)	0.00 - 1.60
EAS-4 Anxiety	0.25±0.30	0.20 (0.00;0.40)	0.00 - 1.40

*EAS - Symptom Assessment Scale.*

Table 3 presents the analysis of the association between variables related to nurses’ work and the four EAS dimensions. A statistically significant association was found between the variable changing profession and the presence of characteristic symptoms of psychoticism, somatization and anxiety among nurses.

**Table 3:** Analysis of the association between the symptomatology of psychological disorders and variables related to factors associated with nurses’ work, Recife, Pernambuco, Brazil, 2020

Variables	EAS Score (General index)	EAS Psychoticism	EAS Obsessiveness/compulsiveness	EAS Somatization	EAS Anxiety
	Mean ± SD	Median (Q1; Q3)	Median (Q1; Q3)	Median (Q1; Q3)	Median (Q1; Q3)
Workload/week (h)					
30- 40 hours	0.45 ± 0.33	0.30 (0.10; 0.60)	0.50 (0.30; 0.90)	0.50 (0.20; 0.70)	0.10 (0.00; 0.50)
>40-60 hours	0.49 ± 0.45	0.45 (0.30; 0.80)	0.50 (0.20; 0.80)	0.40 (0.20; 0.80)	0.20 (0.00; 0.40)
> 60 hours	0.39 ± 0.35	0.50 (0.10; 0.85)	0.50 (0.25; 0.85)	0.20 (0.15; 0.60)	0.10 (0.00; 0.50)
*p* value	0.612 ^*^	0.259 ^**^	0.986 ^**^	0.258 ^**^	0.821 ^**^
Changing profession					
Yes	0.59± 0.55	0.70 (0.30; 1.00)	0.60 (0.40; 0.90)	0.60 (0.40; 0.90)	0.30 (0.10; 0.60)
No	0.41± 0.33	0.40 (0.10; 0.60)	0.50 (0.20; 0.80)	0.40 (0.20; 0.60)	0.10 (0.00; 0.30)
*p* value	0.008 ^A^	0.006 ^B^	0.129 ^B^	0.032 ^B^	0.049 ^B^
Relationships					
1	0.41± 0.40	0.30 (0.10; 0.63)	0.50 (0.28; 0.93)	0.50 (0.20; 0.73)	0.10 (0.00; 0.43)
2 or more	0.47± 0.36	0.40 (0.23; 0.78)	0.50 (0.30; 0.80)	0.40 (0.20; 0.78)	0.20 (0.00; 0.40)
*p* value	0.533^A^	0.145 ^B^	0.907 ^B^	0.851 ^B^	0.844 ^B^
Activity					
Direct care	0.44± 0.35	0.40 (0.18; 0.70)	0.50 (0.20; 0.80)	0.40 (0.20; 0.70)	0.20 (0.00; 0.40)
Indirect care	0.48± 0.39	0.40 (0.20; 0.70)	0.40 (0.30; 0.88)	0.40 (0.23; 0.80)	0.20 (0.00; 0.40)
*p* value	0.561 ^A^	0.734 ^B^	0.646 ^B^	0.422 ^B^	0.976 ^B^
Medication use					
Yes	0.45±0.38	0.40 (0.20; 0.70)	0.50 (0.30; 0.90)	0.40 (0.20; 0.80)	0.20 (0.00; 0.40)
No	0.47±0.35	0.40 (0.20; 0.80)	0.50 (0.20; 0.80)	0.40 (0.30; 0.60)	0.20 (0.00; 0.40)
*p* value	0.859^A^	0.811^B^	0.517^B^	0.487^B^	0.797^B^

* ANOVA;

** Kruskal-Wallis;

A Student’s t;

B Mann-Whitney; SD - standard deviation; EAS - Symptom Assessment Scale.

## DISCUSSION

Based on the results, there was a predominance of females as a workforce, which corroborates statements of the predominance of this sex, making up the profile of the highest percentage of health professionals in many countries^(17)^, especially in Brazil, whose percentages are even more striking^(18)^. Thus, it is argued that females can be influenced by high levels of stress, as a result of discontinuity of relationships between family members and occupational demands, represented by the polarization of functions that the woman, mother and professional are inserted, causing emotional overload when performing these functions simultaneously^(19-20)^.

Thus, a point to be questioned would be the feminization of nursing along with the characteristic of caring for others, labeled as a profession focused on charity, of a domestic nature and little social and economic recognition^(20-22)^. It was addressed in the aspects that involve nurses’ work that the opportunity to reach some level of graduate studies may contribute with less predisposition to levels of stress, dissatisfaction and desire to change jobs or professions^(23)^. It is verified that the investment in professional training, by the institutions, favors in a compensatory way the well-being of these professionals.

The high percentage of professionals who claimed to have more than one bond and extensive working hours expose the living and working conditions assumed by these participants, in the midst of a scenario, sometimes trivialized by public managers and society, but which has been showing constant warning signs.

The fact that there is legality in the capacity to accumulate bonds between health professionals allows exhaustive working hours, due to the search for personal and professional satisfaction and the concept of well-being of each individual. However, the characteristics of the activity developed by nursing professionals, added to the absence of a salary floor that allows distortions between remuneration and workload, corroborate the situation of vulnerability, illness and neglect with their quality of life^(24-25)^.

The data also reflect the feelings and perceptions of professionals regarding quality of life and work practice, when sleep quality is classified. Shift work is inherent to health professionals, for work organization and guarantee of continuity of care; however, there are controversies regarding the shift that favors the highest probability of illness. One of the studies describes the day shift as a predictor of high degree of stress and risk to develop burnout syndrome^(26)^, while others refer to night work as a causal factor related to sleep deprivation, due to the fact of changing the sleep-wake cycle^(27)^.

Given the scenario described, and the psychodynamic theory of workassumptions, it is possible to say that in any work there may be situations and factors that cause suffering. And, in the presence of wear and tear in the work environment, relations tend to weaken, generating individualism, as a result of lack of team cooperation^(28-30)^. Studies describe factors associated with nurses’ working conditions that reflect on the quality of care provided and the directions that the nursing profession has been facing^(31-33)^.

Thus, to understand the process of vulnerability to which nursing professionals are exposed, it is necessary to understand the concept of nursing work and the time taken to carry out this work, regardless of whether they are activities aimed at direct patient care^(34-35)^. This understanding is directly related to work relationships with professionals’ health.

The evidence of the obsessive/compulsive dimension may correspond to the behavior that originates from the safe practices adopted with greater rigor in this pandemic context, in addition to the fear of contamination by COVID-19. Furthermore, nursing professionals were the ones who most added stigma because they were protagonists in the fight against the disease. Conceptually, obsessive compulsive disorder is a chronic neuropsychiatric condition, generated by high loads of anxiety, being characterized by repetitive and intrusive thoughts, accompanied by negative mood or distress, which generate ritualistic behaviors in order to prevent feared events^(36)^.

Considering the findings, other reflections are relevant regarding the probability of acquiring psychological distress related to the historical evolution of nursing in the face of the main epidemics experienced in the country and in the world. Although it represents a milestone that highlighted nursing and made it occupy spaces due to the uniqueness of the activities, the behavior that is established in the scenarios of the profession can trigger suffering and mental disorders, whose chain of relationship with work is widely discussed as one of the main causes of absence^(37)^. It is understood that the situations generated in these scenarios cause stress and compromise both professional performance and interpersonal relationships^(38)^.

With regard to the possibility of changing profession due to the presence of symptoms suggestive of psychological illness, this study revealed the statistically significant association between changing profession and the presence of symptoms of psychoticism, somatization and anxiety. Confronted with this, it is possible to infer that the presence of symptoms indicative of psychological disorders, previously mentioned, can lead professionals to change their profession. However, professionals are not always able to implement such a desire, since some conditions can make it unfeasible or difficult, such as time in the profession, relationship stability, among others. In this regard, the symptoms presented may interfere with the assistance provided by professionals during their workday, and may constitute important risks for themselves, their organization and patients.

Another Brazilian study also found factors associated with the presence of psychopathological symptoms among nurses during the COVID-19 pandemic. The association between workload and psychoticism was found. Moreover, workplace violence, receiving psychological support in the institution in which they work was associated with all EAS-40^(39)^ domains.

These findings reinforce that many nursing professionals are working in working conditions that are not favorable to their health and that can lead them to dissatisfaction and consequently to the desire to change their profession, as observed in this study. Other studies describe professional dissatisfaction at high levels, with remuneration being an influential factor in professionals’ quality of life, but it does not overlap with other stressors discussed in other studies^(40-42)^.

Given the above and the results found in this study, it is possible to observe the presence of psychological symptoms in the various presentations and characterizations associated with thoughts, perceptions, emotions and behaviors that are out of ordinary and interfere with interpersonal relationships^(43)^. The relationships experienced by nursing professionals between the socio-occupational interaction and stress have aroused looks for physical, emotional and psychosocial repercussions over the years. However, this finding, present in several studies^(44-46)^, is consolidated by recognizing that, currently, the psychological domain stands out in the face of other conditioning factors for the illness of nursing professionals^(47-49)^.

At the same time, the influence between the work environment, managers and work processes, associated with personality characteristics, lifestyle, vulnerability and obstinacy to stress, influences workers’ activities, health and performance at work, being the ability to manage these events the greatest challenge related to the response to this coping^(50)^.

### Study limitations

As it is a study in which data collection was carried out with nurses who were in their work environments, this has as a limitation the fact that it was carried out in these spaces, because, on some occasions, filling in the questionnaires had to be momentarily interrupted so that these professionals could meet the work sector demands.

### Contributions to nursing, health, and public policies

Support strategies that improve the professional training of nursing and promote assistance based on the recognition of nursing as a social science, capable of understanding the role of the relationship with society, envisioning human care as something much more complex that also involves individuals who care

## CONCLUSIONS

From the results found in this study, it was possible to verify that thinking about changing profession was associated with the presence of characteristic symptoms of psychoticism, somatization and anxiety among nurses. Since it is a profession characterized by the presence of women, which is culturally intertwined with numerous domestic and family assignments, in addition to the work context, which impose numerous daily challenges, it is pertinent to consider that these results have been influenced by this scenario, which includes most nurses and which, consequently, is impacting their professional satisfaction and their mental health.

When considering the context of the current COVID-19 pandemic, in which the routine of work, family, school and leisure has been completely modified, existing weaknesses reverberate greater suffering in nursing, whose consequences can directly interfere with professionals and the care provided Thinking about promoting quality in care requires, first, thinking about caring for those who care, favoring healthy environments and behavioral changes that provide opportunities for illness.

Thus, investments in better working conditions and welcoming actions in the work environment are fundamental to promote the quality of life at work for health professionals. Therefore, it is urgent to institute to implement regulatory public policies, not focused only on the biological model, but on the integrality of workers whose vulnerability in the nursing scenario has been interfering with the directions of nursing. This is certainly the path to be adopted to prevent symptoms associated with psychological distress in health organizations.
